# Developing a Dissociative Nanocontainer for Peptide Drug Delivery

**DOI:** 10.3390/ijerph121012543

**Published:** 2015-10-09

**Authors:** Patrick Kelly, Prachi Anand, Alexander Uvaydov, Srinivas Chakravartula, Chhime Sherpa, Elena Pires, Alison O’Neil, Trevor Douglas, Mandë Holford

**Affiliations:** 1 Hunter College and The Graduate Center, City University of New York, Belfer Research Building, 413 E. 69th Street, New York, NY 10021, USA; E-Mails: mkelly3@gc.cuny.edu (P.K.); prachiacbr.du@gmail.com (P.A.); auvaydov21@gmail.com (A.U.); sc82@hunter.cuny.edu (S.C.); chhime11@gmail.com (C.S.); pirele92@gmail.com (E.P.); 2 Stem Cell and Regenerative Biology Department, Harvard University, 7 Divinity Ave, Cambridge, MA 02138, USA; E-Mail: alisonloneil@gmail.com; 3 Department of Chemistry, Indiana University, 800 E. Kirkwood Ave., Bloomington, IN 47405, USA; E-Mail: trevdoug@indiana.edu; 4 The American Museum of Natural History, Central Park West & 79th Street, New York, NY 10024, USA

**Keywords:** peptide therapeutics, nanocontainers, drug delivery, P22 bacteriophage, viral capsid, ROMP, controlled disassembly, triggered release, Grubbs catalyst, venom peptides

## Abstract

The potency, selectivity, and decreased side effects of bioactive peptides have propelled these agents to the forefront of pharmacological research. Peptides are especially promising for the treatment of neurological disorders and pain. However, delivery of peptide therapeutics often requires invasive techniques, which is a major obstacle to their widespread application. We have developed a tailored peptide drug delivery system in which the viral capsid of P22 bacteriophage is modified to serve as a tunable nanocontainer for the packaging and controlled release of bioactive peptides. Recent efforts have demonstrated that P22 nanocontainers can effectively encapsulate analgesic peptides and translocate them across blood-brain-barrier (BBB) models. However, release of encapsulated peptides at their target site remains a challenge. Here a Ring Opening Metathesis Polymerization (ROMP) reaction is applied to trigger P22 nanocontainer disassembly under physiological conditions. Specifically, the ROMP substrate norbornene (5-Norbornene-2-carboxylic acid) is conjugated to the exterior of a loaded P22 nanocontainer and Grubbs II Catalyst is used to trigger the polymerization reaction leading to nanocontainer disassembly. Our results demonstrate initial attempts to characterize the ROMP-triggered release of cargo peptides from P22 nanocontainers. This work provides proof-of-concept for the construction of a triggerable peptide drug delivery system using viral nanocontainers.

## 1. Introduction

For much of the twentieth century, “drug discovery and development” was synonymous with the identification and synthesis of biologically active small molecules derived from natural products or created through rational-design processes. However, since 1980 the number of new chemical entities (NCEs) registered each year with the FDA has remained more or less constant, despite the proliferation of newly identified drug targets and the considerable financial and scientific resources devoted to drug discovery [1,2]. Bioactive peptides, which are very potent, highly selective, and do not produce toxic metabolites upon degradation, are a promising alternative to small molecules for drug discovery and development [1,3]. There are numerous natural reservoirs of bioactive peptides, such as the venom arsenal of predatory marine snails, spiders, and other organisms, that have yet to be thoroughly explored and characterized [4,5,6,7,8]. Ziconotide (Prialt®), a peptide drug first identified in the venom of the marine snail *Conus magus*, is the first non-opioid analgesic and is currently used to alleviate chronic pain in HIV and cancer patients [8,9,10].

The primary obstacle to the widespread use of bioactive peptides has been their unfavorable pharmacokinetic profile. Unstructured peptides are degraded by endogenous proteases and are thus relatively unstable *in vivo* [11]. Additionally, most peptides, with the exception of specialized cell-penetrating peptides, cannot cross the blood-brain barrier (BBB) [12]. However, peptide therapeutics derived from venomous organisms exhibit improved pharmacokinetic profiles, in part due to the structural integrity provided by disulfide bonds. Ziconotide, for example, is stabilized by three disulfide bonds that confer increased resistance to endogenous proteases. The structural stability of ziconotide ensures prolonged relief when it is administered as a pain therapy [8,9,10]. However, ziconotide does not cross the BBB, and must be administered by intrathecal injection [10]. An alternative to intrathecal injection for the administration of ziconotide, in which the peptide was encapsulated in a viral nanocontainer and delivered across the BBB via a trojan horse strategy, was recently described [12]. 

Packaging peptides in a nanocontainer delivery system is an effective strategy for improving their pharmacokinetic profile, as the nanocontainer can protect the peptide during transport and release at the site of its molecular target. The list of macromolecules that have been investigated for their potential as delivery vectors is extensive and diverse. It includes polymeric nanoparticles [13], micelles [14], liposomes [15], DNA origami structures [16], and protein cages such as viral capsids (with the infectious genome removed) and ferritins [17,18]. Regardless of the type of macromolecule used, nanocontainer delivery systems generally consist of three steps: packaging of the pharmacological agent, targeting of the loaded nanocontainer to the appropriate site *in vivo*, and disassembly or release of the active compound. While strategies for packaging and targeting nanocontainers have been extensively studied, controlled release of the encapsulated drug remains problematic [12,19,20]. In large part, hurdles to nanocontainer disassembly persist because the release mechanism needs to function under physiological conditions of moderate temperature, neutral pH, and aqueous environment.

Here, we present a strategy for inducing disassembly of a nanocontainer derived from the viral capsid of the *Salmonella typhimurium* bacteriophage P22 under physiological conditions. The P22 capsid is a T = 7 icosahedral lattice composed of 420 copies of P22 coat protein (CP) that self-assemble in the presence of approximately 300 copies of the P22 scaffold protein (SP) [21]. A truncated version of the SP, consisting of the last 66 C-terminal residues, is sufficient for self-assembly of the procapsid [22]. The Douglas group recently developed a system for packaging arbitrary gene products into the P22 capsid shell by using standard recombinant methods to create a fusion protein consisting of an arbitrary cargo protein joined to the P22-SP C-terminus by a thrombin cleavage site [23]. Various gene products have been loaded into the P22 capsid in this manner, including the fluorescent proteins EGFP and mCherry, the enzyme alcohol dehydrogenase D, and ziconotide [12,23,24]. 

**Figure 1:**
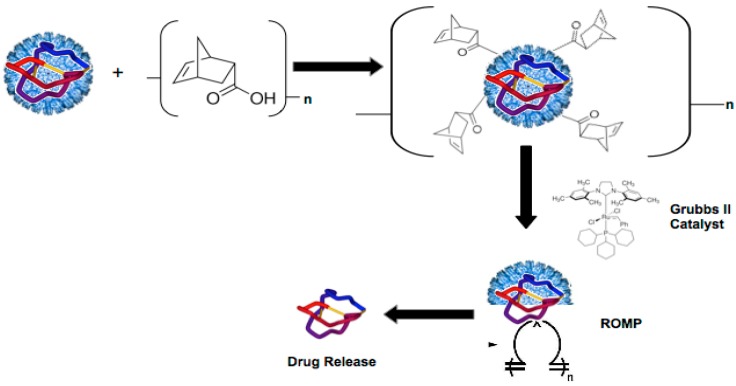
Creating a dissociative viral nanocontainer for peptide drug delivery. A viral nanocontainer is conjugated to strained olefins that undergo a ring opening polymerization reaction (ROMP) in the presence of Grubbs II Catalyst. The ROMP reaction disrupts the architecture of the nanocontainer, resulting in disassembly and release of the encapsulated peptide cargo.

Our strategy for triggered disassembly of the P22 nanocontainer employs Ring Opening Metathesis Polymerization (ROMP), an olefin metathesis reaction that is known to proceed under physiological conditions in the presence of a ruthenium catalyst (Grubbs Catalyst) [25,26]. The ROMP disassembly strategy is desirable as it can be triggered to release the cargo in response to a defined stimulus, which will provide temporal control over the pharmacokinetics of the encapsulated drug [27]. Specifically, we conjugated the ROMP substrate norbornene (5-Norbornene-2-carboxylic acid) to the exterior of loaded P22 nanocontainers and used a 2nd Generation Grubbs Catalyst (Grubbs II catalyst) to trigger the ROMP reaction. Our results indicate that the ROMP reaction disrupts the P22 nanocontainer architecture, which could lead to release of the encapsulated cargo (Figure 1).

## 2. Materials and Methods

### 2.1. Construction of P22-GFP Nanocontainers 

**Figure 2:**
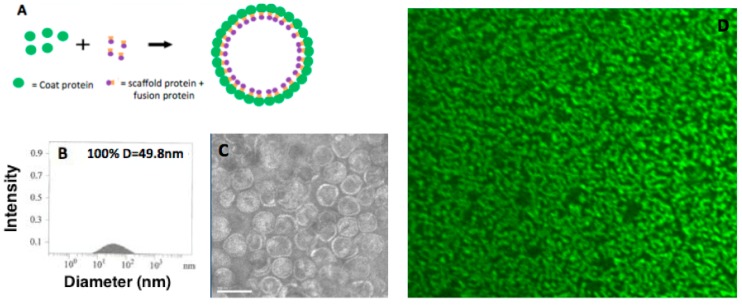
Construction of the P22-GFP Nanocontainer. (**A**) Incorporation of cargo protein into P22 capsid. When the P22 coat protein (green circles) is co-expressed with an engineered scaffold protein-cargo protein construct (orange squares and purple circles, respectively), the nanocontainer self-assembles with the coat protein on the exterior and the scaffold and fusion proteins on the interior. Here, the cargo protein is GFP. (**B**) Dynamic Light Scattering (DLS) of unconjugated P22-GFP nanocontainers reveals spherical entities with a mean diameter of 49.8 nm. (**C**) TEM (Transmission Electron Microscopy) image of P22 nanocontainers. The assembled capsids are homogeneous in size, shape, and packaging. Scale bar = 100 nm. (**D**) Confocal fluorescence microscope image of P22-GFP at 100× magnification confirms the presence of GFP.

P22-GFP nanocontainers were constructed as described in O’Neil *et al*. [23] (Figure 2A). Briefly, BL21 *E. coli* were transformed with a plasmid containing the genes for the P22 coat protein and an engineered GFP-scaffold protein fusion. Transformed *E. coli* were grown to OD_600_ = 0.6 and induced with isopropyl β-D-1-thiogalactopyranoside (IPTG). Cells were harvested and lysed, and clarified lysates were subject to ultracentrifugation over a 35% sucrose cushion. Viral pellets were further purified over a sephacryl (S-500) size-exclusion column (GE Healthcare). Detection of GFP protein was achieved by electrospray ionization mass spectrometry (45,515 Da for EGFP-SP141 fusions) to confirm successful encapsulation inside P22 nanocontainers.

### 2.2. Confocal Fluorescence Microscopy Characterization of P22-GFP Nanocontainers

Spinning disc confocal microscopy was carried out on a Perkin Elmer UltraView ERS instrument using a GFP filter. Samples of P22-GFP were diluted 16x with nanopure water, mounted onto a slide, and examined at 100× magnification. Images were produced with Volocity 3D Image analysis software.

### 2.3. Conjugation of Norbornene to P22-GFP Nanocontainers 

To conjugate norbornene to surface-exposed lysine residues on the P22 bacteriophage capsid, 1.4 mg of 1-ethyl-3-(3-dimethylaminopropyl) carbodiimide (EDC) and 1.6 mg *N*-hydroxysuccinimide (sulfo-NHS) were added to 1 mL of a (1 mg/mL) solution of 5-norbornene-2-carboxylic acid in 0.1M phosphate buffer (pH 6.0) (all reagents were purchased from Sigma-Aldrich). The solution was allowed to react at room temperature with intermittent vortexing. After 30 minutes, 0.1 M dibasic phosphate stock was added dropwise to raise the pH to 7.5. To this activated 5-Norbornene-2-carboxylic acid solution was added 250 μL of (13.2 mg/mL) P22-GFP in PBS. After reacting at room temperature overnight on a shaker at 250 rpm, the product was concentrated by means of a 10 KDa MWCO centrifugal filter device (Amplicon) and resuspended in pH 7.0 phosphate buffer. 

### 2.4. MS Characterization of P22-GFP-Norbornene Nanocontainers

Mass Spectrometry (MS) analyses were carried out on a 6520 Accurate Mass QTOF LC/MS system (Agilent Technologies). P22 nanocontainers conjugated with 5-Norbornene-2-carboxylic acid (1 μL, 1.0 mg/mL) were injected into an Agilent Eclipse Plus C18 column and eluted with 99.9% ACN, 0.1% formic acid. Spectra were produced using Agilent MassHunter Qualitative Analysis software. 

### 2.5. Ring Opening Metathesis Polymerization (ROMP) of P22-GFP-Norbornene Nanocontainers 

Two samples of (2 mg/mL) P22-GFP-Norbornene were transferred to microfuge tubes in aliquots of 20 μL. To the first (control) sample was added 1.7 μL of nanopure H_2_O. To the second (experimental) sample was added 1.7 μL of 0.01% (w/v) (10 mol%) Grubbs II Catalyst (Sigma-Aldrich). Samples were vortexed and allowed to react 24 hours at room temperature.

### 2.6. Transmission Electron Microscopy (TEM) Characterization of P22-GFP Nanocontainers and Post-ROMP P22-GFP-Norbornene Nanocontainers

TEM images were produced on a JEOL JEM 2100 instrument with an acceleration voltage of 200 kV. Samples were applied to a carbon-coated copper grid and stained with uranyl acetate solution, then dried at room temperature. Images were processed and analyzed using ImageJ software.

### 2.7. DLS Characterization of P22-GFP Nanocontainers

Dynamic light scattering (DLS) measurements were recorded using a PD2000DLS instrument (PDDLS/Cool Batch 90T, Precision Detectors). Nanocontainers at a concentration of 1 mg/mL in pH 7.2 PBS were filtered through a 0.2 μm syringe filter and transferred to a 0.5 × 0.5 mm glass cuvette for DLS analysis. 

### 2.8. P22-GFP Heat-Activated Disassembly Monitored by Native Agarose Gels

P22-GFP-Norbornene samples were divided into eight aliquots of 15 μL each and heated for 10 minutes under a thermal gradient. Specifically, the first aliquot was heated at 50 °C, with 5 degrees added for each subsequent aliquot, so that the final aliquot was heated at 85 °C. Samples were allowed to cool, mixed with loading buffer (40% glycerol, bromophenol blue) and loaded into the wells of a 1.0% native agarose gel with unheated P22-GFP and P22-GFP-Norborne controls. Gels were run at 65 V for 2.5 hours in TAE buffer (40 mM tris, 20 mM acetic acid, 1 mM EDTA), then stained with coomassie blue and destained with acetic acid and methanol. Images were produced using a Foto/Analyst FX imaging system (Fotodyne).

## 3. Results

### 3.1. Characterization of P22-GFP Nanocontainers

The integrity of P22-GFP nanocontainers was determined using dynamic light scattering (DLS), TEM, and spinning disk confocal fluorescence microscopy. DLS analysis detected spherical nanocontainers with a mean diameter of 49.8 nm (expected mean diameter 50.0 nm) [23]. TEM images revealed capsids that are homogeneous in size and shape (Figure 2B and Figure 2C). Spinning disk confocal fluorescence microscopy at 100× magnification and 16× dilution revealed a field of bright green dots, indicating the successful encapsulation of GFP. DLS, TEM, and confocal fluorescence microscopy characterization, together with prior MS data [23] confirmed the construction of P22-GFP nanocontainers. 

### 3.2. Characterization of Norbornene-Conjugated P22-GFP Nanocontainers 

**Figure 3:**
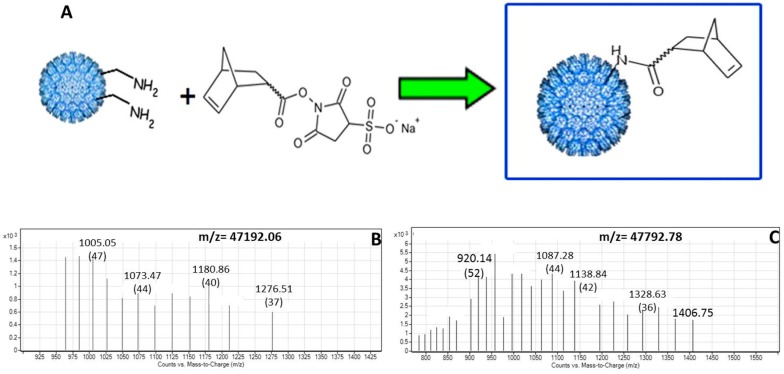
Conjugation of Norbornene-COOH to the Capsid Surface. (**A**) Norbornene-COOH is activated with EDC and sulfo-NHS, leading to the formation of an amide bond with the ε-NH_2_ of surface-exposed lysine residues. (**B**) Mass spectrum of unmodified capsid coat protein (m/z = 47,192). (**C**) Mass spectrum of coat protein subunit conjugated with five norbornene subunits (m/z = 47,792).

The P22-GFP-norbornene conjugation reaction was monitored by MS analysis. Compounds with masses corresponding to that of the P22 coat protein (expected mass 46,596 Da, observed mass 47,192 Da) plus an integral number (between 2 and 6) of 120 Da adducts was detected (Table 1). The 120 Da mass corresponds to a 5-norbornene-2-carboxylic acid moiety conjugated to a free amine to form an amide bond (Figure 3). A weighted average of the number of norbornene adducts per coat protein monomer (with the weight determined by the relative abundance by volume of each compound cited in Table 1) suggests there are slightly more than four norbornene moieties per coat protein monomer, or approximately (4 norbornene adducts per monomer) × (420 coat protein monomers per nanocontainer) equates to 1700 norbornenes per nanocontainer. Although the P22 coat protein contains 19 lysine residues that could potentially couple with an activated 5-Norbornene-2-carboxylic ester, not all of these are surface-exposed, which would explain why not all 19 lysines are conjugated with norbornene. 

**Table 1:** Compounds detected through MS analysis of norbornene-conjugated P22-GFP nanocontainers. Seven P22-Norbornene compounds were detected by MS analyses. The unconjugated coat protein had a baseline observed mass of 47,192 Da (expected mass = 46,596 Da). Each additional conjugated norbornene adds an average of 120 Da. to the baseline mass. The volume-weighted average is 4.12 norbornenes per coat protein monomer.

Compound	Mass	Vol %	No. of Norbornenes
5	47,792.2998	26.8	5
4	47,672.4353	24.17	4
3	47,552.3638	20.9	3
2	47,672.0044	12.43	4
6	47,911.832	5.81	6
1	47,432.2401	5.24	2
7	47,911.4899	3.95	6

### 3.3. TEM Characterization of P22-GFP-Norbornene ROMP Reaction

**Figure 4:**
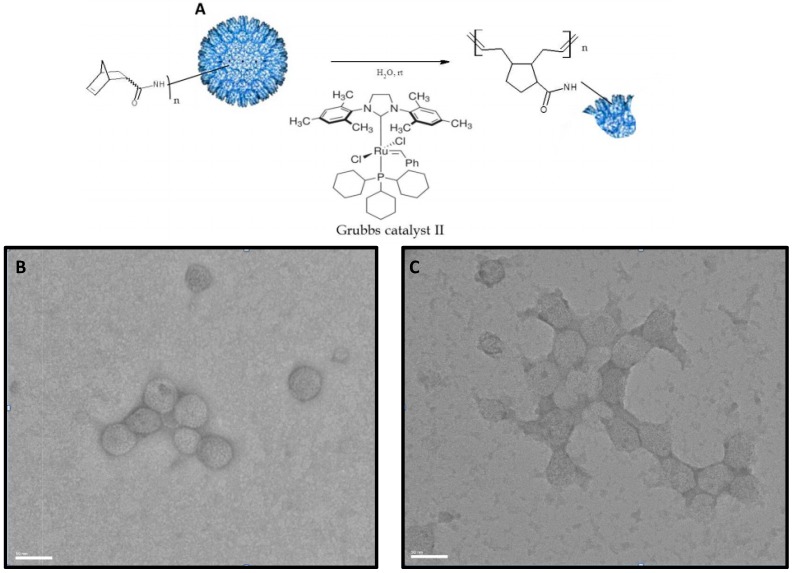
Disassembly of P22 nanocontainer using Grubbs Catalyst-activated ROMP. (**A**) Reaction scheme of ROMP (Ring Opening Metathesis Polymerization) reaction. P22 nanocontainers conjugated with norbornene are treated at room temperature under physiological conditions with Grubbs II Catalyst, which triggers polymerization of norbornene and disruption of nanocontainer conformation. TEM of nanocontainers before ROMP reaction (**B**), and after ROMP (**C**), illustrate the morphological change. P22-norbornene nanocontainers treated with 10 mol% Grubbs II catalyst exhibit strained and distorted morphologies when compared with untreated P22-GFP-Norbornene nanocontainers. Treated nanocontainers also exhibit robust bridge structures, suggesting that the ROMP reaction is occurring at both intra- and inter-nanocontainer interfaces. Scale bar = 50 nm.

The integrity of the P22 nanocontainers before and after conjugation with norbornene and treatment with Grubbs II catalyst was monitored by TEM. TEM revealed that P22-GFP-Norbornene capsids treated with 10 mol% Grubbs II catalyst exhibited strained and distorted morphologies when compared with untreated P22-GFP-Norbornene (Figure 4). Treated capsids also exhibited robust bridge structures at inter-capsid interfaces. The ROMP reaction was functional under experimental conditions. 

### 3.4. Characterization of Heat-Induced Dissociation of P22 Nanocontainer

**Figure 5:**
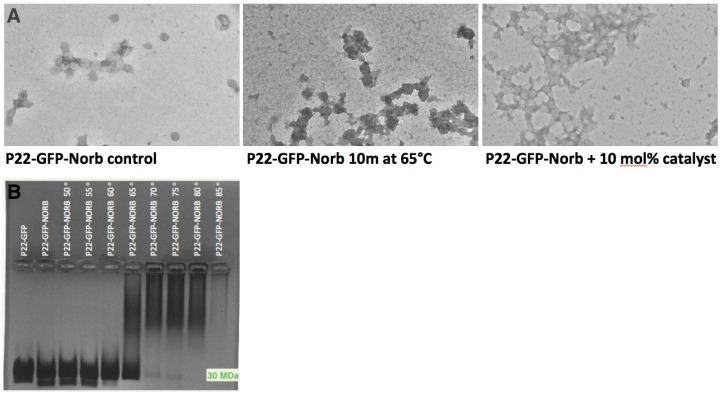
Heat-induced disassembly of P22 nanocontainers *vs*. ROMP-triggered morphological distortion of P22-GFP-norbornene nanocontainers. (**A**) Heating of P22-GFP nanocontainers at 65 °C for 10 m results in a rough, distorted texture that indicates the beginning of capsid dissolution. P22-GFP-Norb nanocontainers treated with 10 mol% Grubbs II catalyst exhibit a similar pattern of distortion. (**B**) Native 1% agarose gel of heated P22-GFP-Norb nanocontainers shows the onset of disassembly after 10 m at 65 °C. All TEM images at 20k magnification.

P22 nanocontainers are known to undergo morphogenesis when heated. The nanocontainers expand to a diameter of 64 nm when heated at 65 °C and shed the coat protein pentons at each of the 12 fivefold axes of symmetry when heated at 75 °C. Heating above these temperatures causes disassembly [23,28]. Native agarose gel characterization illustrated that P22-GFP-Norbornene nanocontainers begin to dissociate after 10 minutes in the 60–70 °C range, as indicated by the disappearance of the 30 MDa band (Figure 5). The streaking towards the top of the gel in lanes, corresponding to temperatures of 65 °C and above, is likely due to the formation of large amorphous aggregates, which are visible in the background of TEM images of samples of P22-GFP-Norbornene heated for 10 minutes at 65 °C (Figure 5A). Significantly, TEM images of P22-GFP-Norbornene treated with 10 mol% Grubbs II catalyst and TEM images of P22-GFP-Norbornene heated for 10 minutes at 65 °C show similar patterns of morphological distortion, suggesting dissociation of the nanocontainers (Figure 5A). 

## 4. Conclusions

Delivering peptide drugs to their site of action in a controlled manner remains a significant challenge. Here we describe a drug delivery system that encapsulates a peptide cargo within a viral nanocontainer and employs a catalyst-driven ROMP reaction to trigger release of the encapsulated cargo. Specifically, activated 5-Norbornene-2-carboxylic acid was conjugated to surface-exposed lysine residues on the P22 bacteriophage procapsid in a yield of approximately four norbornene moieties per P22 coat protein monomer. Treatment of the P22-GFP-Norbornene nanocontainer with Grubbs II catalyst precipitated a ROMP reaction that distorts the morphologies of individual nanocontainers. These results illustrate a method for inducing conformational changes in engineered P22 bacteriophage nanocontainers that will enable the controlled release of loaded peptide cargos.

Recent studies have illustrated that viral capsid nanoplatforms, such as the P22 capsid used in this study, are not toxic in cell lines, nor when administered to mice [12,29]. In particular, the Kaiser *et al.* study provided a baseline for protein nanoplatform degradation and clearance *in vivo.* The Kaiser study found that single injections of naïve and immunized mice with representative protein cage nanoplatforms derived from the Cowpea chlorotic mottle virus (CCMV) and the heat shock protein (Hsp) of the hyperthermophilic archaeon *Methanococcus jannaschii* did not lead to overt toxicity. Protein cages were broadly distributed throughout most tissues and organs, were rapidly excreted within 24 hours, and did not exhibit long-term persistence in tissue or organs. While a comprehensive analysis has yet to be performed on the P22 capsid, these results suggest that the protein cage of P22 capsid may also be safe for biomedical applications. In addition, preliminary reports indicate that the polynorbornene produced by the ROMP disassembly reaction is not cytotoxic [30].

The Grubbs catalyzed ROMP strategy for nanocontainer disassembly that we have pursued here is novel. Previous strategies for nanocontainer disassembly have required the manipulation of environmental conditions such as pH and temperature that cannot be readily controlled *in vivo* [11]. Our ROMP-based method builds upon the resurgence in material and natural product chemistry applications that followed the introduction of solvent-tolerable catalysts such as the ruthenium-based Grubbs catalyst in the 1990s and 2000s. The Grubbs catalyst not only accelerated metathesis reactions to a physiologically relevant timescale (in many cases a matter of minutes), but also allowed for the use of water-based solvents and proved effective at initiating metathesis reactions in minimal concentrations [31]. In addition, the water-compatible properties of the ROMP reaction suggests that the catalyst could be administered to patients via intravenous drip therapy, similar to saline.

While we have successfully demonstrated the ROMP reaction, there are several questions that need to be addressed to make this a viable method *in vivo*. For example, the Grubbs II catalyst applied is ruthenium-based, and ruthenium (Ru) is mildly toxic [32,33]. However, the amount of Grubbs II catalyst needed to initiate a ROMP reaction is relatively small. Here we applied 10 mol% Grubbs II catalyst, however, other studies report effective catalysis in water with amounts as low as 2 mol% [34]. Additionally, there have been efforts to produce ROMP catalysts based on elements with reduced toxicity, such as molybdenum (Mo) and tungsten (W) [35,36]. Replacing Ru with Mo or W would extend the potential application of *in vivo* ROMP nanocontainer dissociation. 

Finally, more recent iterations of ROMP catalysts allow for photoactivated reactions that are initiated when ROMP monomers are exposed to UV-visible light (~365–420 nm) in the presence of a photolabile catalyst [37]. One recent study used light at similar wavelengths (380–500 nm) to trigger a photoswitchable affinity tag targeting potassium channels in cultured cerebral cells. Results from the same study indicated that brain tissue did not significantly impede light penetration using 380–500 nm [38]. An alternative strategy for triggering a photoactivated ROMP reaction within the CNS would be to use a red-shifted photoswitchable catalyst, since near-IR radiation can more effectively penetrate tissue. Photoactivated ROMP could thus provide a trigger mechanism for exerting precise spatial and temporal control over the release of encapsulated drug cargoes. Together with our findings, these recent studies pave the way for the development of a ROMP-triggered P22-based nanocontainer system for the controlled delivery of peptide therapeutics.
